# Editorial: Music Training, Neural Plasticity, and Executive Function

**DOI:** 10.3389/fnint.2020.00041

**Published:** 2020-08-07

**Authors:** Paul J. Colombo, Assal Habibi, Claude Alain

**Affiliations:** ^1^Department of Psychology, School of Science and Engineering, Tulane University, New Orleans, LA, United States; ^2^Brain and Creativity Institute, Department of Psychology, University of Southern California, Los Angeles, CA, United States; ^3^Rotman Research Institute, Department of Psychology, University of Toronto, Toronto, ON, Canada

**Keywords:** executive function, cognitive control, neural plasticity, music training, prefrontal cortex, neural oscillation, absolute pitch, longitudinal study

Music training is rapidly emerging as an important model system for investigating experience-dependent brain plasticity, and also as the basis for robust sensory, motor, and cognitive therapeutic interventions. This special topic contains articles describing theoretical and methodological advancements that further our understanding of relationships between musicianship, music training and executive function, and many of the articles delve into the neural mechanisms that drive these relationships. It is well-established that music training is associated with sensory, motor, and cognitive benefits. As described in the articles in this collection, investigators are characterizing how elements of musical abilities (e.g., absolute pitch and relative pitch processors) and training (e.g., amateur vs. professional), are related to components of executive function including inhibitory control, working memory, and cognitive flexibility. In several reports, new analytical methods are being implemented to probe neural mechanisms of plasticity associated with music training and performance.

## Music Training Interventions Improve Executive Functions in Relation to Brain Plasticity: Childhood Development and Older Adults

Systematic manipulations of musical experience are the key to making causal statements about their effects on sensory, motor, and cognitive outcomes, and several investigators used this approach. For example, Dubinsky et al. report that short-term choir singing improves speech-in-noise perception and pitch discrimination among older adults with hearing loss, and improvement is related to the strength of the frequency following response, a neural representation of auditory stimuli. In another study, older adults received different types of musical experience, including music listening, piano training, or percussion training. Both of the active music groups outperformed the listening group in bimanual synchronization and visual scanning/working memory, and piano training significantly improved motor synchronization skills in comparisons with the percussion training or listening groups (Bugos). Frischen et al. also tested effects of different types of music training by assessing several measures of executive functions, then randomly assigning preschoolers to rhythm-based or pitch-based music training. Inhibition improved from pre- to post-test among children who received rhythm-based training, but not pitch-based training or a sports control, and similar numeric differences were found for measures of set shifting and visuospatial working memory. Taken together, these studies demonstrate that music training causes improvement of sensory, motor, and cognitive control processes among children and older adults, and are not merely reflections of pre-existing neurocognitive differences. Moreover, they begin to dissociate components of musical training that may lead to improvement of specific cognitive processes. With regard to very early childhood development, Loewy and Jaschke identify how parameters of music such as timing, timbre, and repetition may influence cognitive development and neural plasticity in therapeutic interventions with neonates. The advancements noted above are bolstered by results of longitudinal studies of musically trained children in which working memory (Saarikivi et al.) and inhibitory control (Hennessy et al.) were assessed in relation to years of training. Musically trained children outperformed controls on the trails A and B tests, and forward digit span, but not backward digit span, suggesting that music training may selectively influence working memory capacity and maintenance more than with updating (Saarikivi et al.). Hennessy et al. report that children with 3 years of music training chose a larger, delayed reward in place of a smaller, immediate reward when compared to children without music training, indicating enhancement of delayed-gratification measures of inhibition. In the flanker task, children in the music group improved their performance accuracy parallel to increasing years of training, while such improvements were not observed in in the groups without music training. The groups were matched at the onset of the study to have no differences among them in cognitive capacities, providing evidence that systematic music-based training accelerates development of inhibitory control in children.

## Comparisons Between Adult Musicians and Non-Musicians Reveal Mechanisms of Enhanced Executive Functions

The articles cited above provide new evidence for behavioral and neural mechanisms of cognitive benefits caused by music training during early childhood development, and among older adults. A parallel approach, taken by several contributors, compared behavioral performance on control processing tasks and neural measures of activity among adult musicians and non-musicians (Manno et al.; Sharp et al.; Sharma et al.; Coll et al.; Criscuolo et al.). This approach yielded insights into the mechanisms by which musical experience may enhance cognitive processes. For example, musician advantages in emotion processing are related to greater use of temporal fine structure information (Manno et al.), and to better identification of complex emotional content in both the auditory and tactile sensory modalities (Sharp et al.). Sharma et al. presented non-musicians, and musicians with either relative or absolute pitch, with three different versions of an auditory Stroop task to measure conflict resolution. The pitch-label association ranged from simple semantic associations (i.e., “Low” or “High”) to intermediate verbal encodings with no obvious semantic properties (i.e., “Doh” or “Soh”) to more abstract semiotic associations (i.e., “C” and “G”). The neural activity indexing conflict detection for abstract pitch label (i.e., musical notation) was present only in musicians with absolute pitch, consistent with a strong automaticity in retrieving the pitch-label association. Coll et al. also showed greater brain electrical source activity in left temporal junctions in musicians with absolute pitch, which could play a part in the automatic retrieval of pitch-label associations. In addition, Criscuolo et al. show that associations between musical experience and enhanced cognitive abilities, which are frequently reported among children, are also evident among adults after controlling for potential confounding variables including age, education, socio-economic status, and personality variables. Musicians show higher general intelligence, verbal intelligence, working memory, and attention than non-musicians, while amateur musicians score in between. It is notable that the reported correlations between years of musical playing and cognitive abilities support the hypothesis that musical practice is associated with intelligence and executive functions.

## Neural Mechanisms of Sensory and Cognitive Processing Among Musicians

In addition to innovative behavioral and cognitive assessments, several investigators reported neurophysiological effects of engagement in auditory and musical processes among musicians. In one striking example, Müller and Lindenberger developed a method to study intra- and inter-brain synchronization, or so-called extended hyper-brain networks, by measuring phase synchronization between transformed acoustic recordings of guitar signals and raw EEG signals of guitarists freely improvising in duet. Of importance, this form of time-frequency analysis incorporates the dynamic interaction of both musicians' production and responses to music. As reviewed by, Yurgil et al. the study of neural oscillations is ideal for investigating neural processes occurring over durations of time spanning seconds to minutes, and particularly suitable for investigating components of executive function such as temporal stages of working memory. In addition to studies of neural oscillations in relation to musical experience and executive functions, event-related potentials yield more temporally discrete information about cortical functions. As an example, Matsuda et al. report a dissociation of auditory cortical potentials in relation to music training and absolute pitch, indicating distinct stages of pitch processing in addition to hemispheric specialization of auditory cortical functions. Salvari et al. examined the functional connectivity among regions of the auditory pathway in processing natural, musical, and artificial non-speech sounds, by means of MEG. The different categories of sounds produced differences in activation and interconnection in prefrontal areas, anterior-superior temporal gyrus, posterior cingulate cortex, and supramarginal gyrus, demonstrating an extended brain network of human-made musical and artificial sounds when compared to natural sounds. A summary of behavioral, neuroimaging, and neurophysiological studies in musicians, non-musicians, and clinical populations is taken up in the Perspective article by Koshimori and Thaut in which they discuss potential for executive function and attentional processing stimulation and neurorehabilitation.

## Summary

Taken together, the articles in this Research Topic represent significant advances in our understanding of relationships between musical training and control processes that comprise executive function. They illustrate the range of research questions that are being addressed by application of neuroscientific methods and technology to the universal cultural phenomenon of musical experience. They represent development of a model system for investigating long-term experience-dependent neuronal plasticity, and they bring into focus components of musical experience that may be targeted for specific therapeutic interventions aiming to benefit individuals across their lifespan.

## Author Contributions

All authors drafted, revised, and approved the final version of the manuscript for submission.

## Conflict of Interest

The authors declare that the research was conducted in the absence of any commercial or financial relationships that could be construed as a potential conflict of interest.

